# Adult Intestinal Malrotation With Congenital Transverse Meso-Colic Internal Hernia: An Infrequent Cause of Small Bowel Obstruction

**DOI:** 10.7759/cureus.63063

**Published:** 2024-06-24

**Authors:** Shariful Islam, Avidesh H Mahabir, Richard Harkissoon, Malini Ramnarine, Patrick Harnarayan

**Affiliations:** 1 General Surgery/Oncoplastic Breast Surgery, San Fernando General Hospital, San Fernando, TTO; 2 General Surgery, San Fernando General Hospital, San Fernando, TTO; 3 Clinical Surgical Science, The University of the West Indies, St. Augustine, TTO

**Keywords:** transverse mesocolic hernia, small bowel obstruction, midgut volvulus, congenital internal hernia, adult intestinal malrotation

## Abstract

Adult intestinal malrotation along with congenital transverse-mesocolic internal hernia causing small bowel obstruction is extremely rare. Most of these patients don't have any obvious clinical symptoms. Only a few cases have been documented in the English literature. We present the unique case of a 43-year-old male without any prior surgical history who presented with nonspecific abdominal pain and was diagnosed with malrotation of the small intestine by computed tomography (CT) scan and underwent exploratory laparotomy found to have internal herniation through the transverse-mesocolon. The patient underwent an emergency laparotomy; a Ladd’s procedure and repair of the hernial orifice were performed. This case highlights the association of adult intestinal malrotation with internal hernias and small bowel obstruction; it also explores the importance of timely diagnosis and adequate management of this condition.

## Introduction

Adult intestinal malrotation with a congenital internal hernia is a rare but well-known cause of small bowel obstruction in the pediatric population [1], however, its incidence is growing amongst the adult population (0.2% in live birth vs 0.2%-0.5% in the adult population) [2,3]. In addition to this, congenital internal hernias are rare inborn pathologies, which may also result in bowel obstruction with potential subsequent strangulation [4,5].

We, therefore, present a case of an adult male with a virgin abdomen with an index presentation of small bowel obstruction secondary to an internal hernia and accompanying malrotation managed successfully via laparotomy. This internal hernia itself is a rare pathology of a trans-ascending mesocolic hernia.

This case highlights the association of adult intestinal malrotation with internal hernias and small bowel obstruction; it also explores the importance of timely diagnosis and adequate management of this condition.

## Case presentation

A 43-year-old man presented to the emergency department with a five-day history of periumbilical abdominal pain, nausea, vomiting, diarrhea, and abdominal distension following consumption of reheated rice. The patient also gave a history of rapid weight loss over the last three months. He had no prior medical or surgical history.

**Figure 1:**
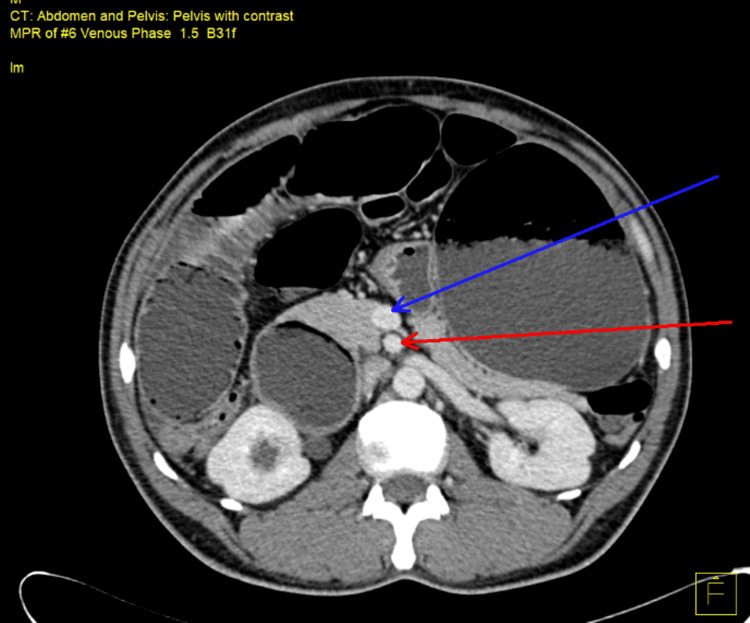
Axial view of venous phase of contrast enhanced CT scan of abdomen and pelvis showing superior mesenteric vein (blue arrow) and superior mesenteric artery (red arrow)

**Figure 2:**
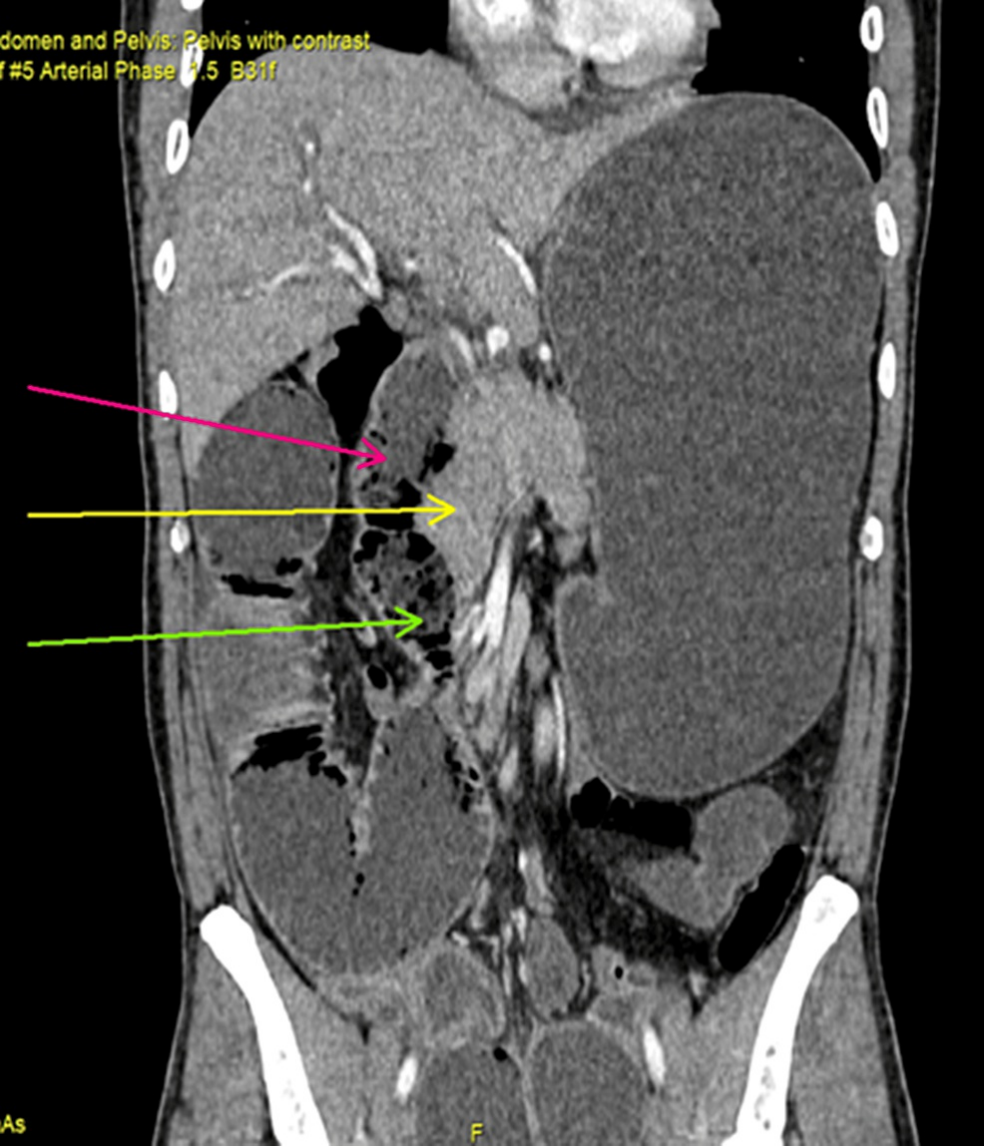
Coronal view of venous phase of contrast enhanced CT scan of abdomen and pelvis showing second part duodenum (pink arrow), uncinate process of pancreas (yellow arrow), duodenojejunal flexure of the right of abdomen (green arrow)

On examination, the patient was hemodynamically stable with a distended, soft, non-tender abdomen. His laboratory investigations were unremarkable. Plain abdominal radiographs showed a distended small bowel. A contrast-enhanced computed tomography (CECT) of the abdomen and pelvis revealed malrotation of the small bowel. The superior mesenteric artery (SMA) was noted posterior to the superior mesenteric vein (SMV) (Figure 1) with a Whirlpool sign of the small bowel mesentery (Figure 2). The small bowel was noted to be viable based on CECT findings. The patient underwent an emergent laparotomy.

**Figure 3:**
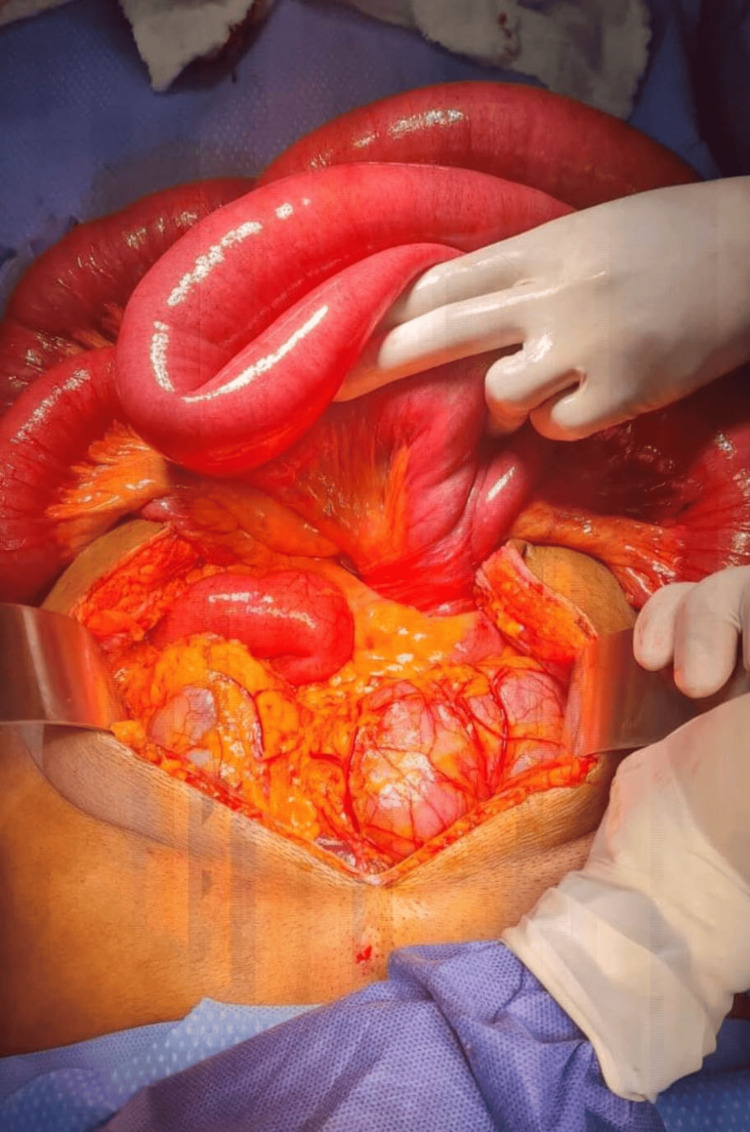
Intraoperative photo showing volvulus of the midgut (inferior aspect of image cranially)

**Figure 4:**
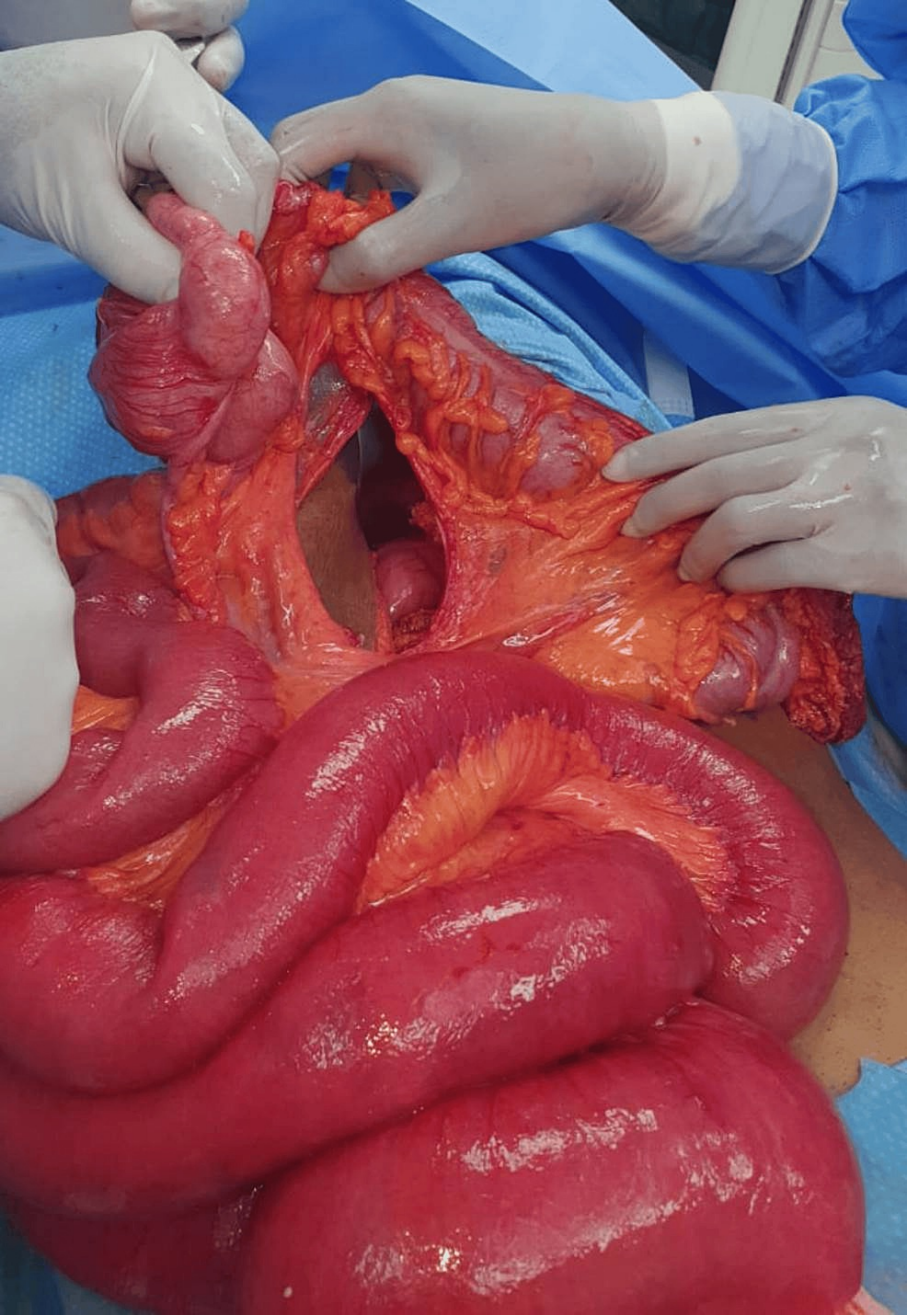
Image showing trans-ascending mesocolon internal hernia.

At laparotomy, a distended but viable small bowel was noted with the caecum in the right upper quadrant with Ladd’s bands indicating an intestinal malrotation (Figure 3). Along the axis of the SMA there was a clockwise midgut volvulus and a small bowel (proximal jejunum) was also noted to be entrapped within a 10cm internal hernia defect within the mesentery of the ascending colon (Figure 4).

No other pathology was identified. The volvulus was de-rotated, Ladd’s bands were taken down and the bowel was placed in a nonrotated position. The mesenteric defect was closed with 2-0 polyglactin. His post-op stay was unremarkable and he was discharged home on the third postop day. Outpatient follow-up has been uneventful.

## Discussion

Intestinal rotation can be defined as the aberrant rotation of the embryonic gut resulting in abnormalities of rotation and fixation. Normal embryological progression dictates that between the fifth to 12th weeks of development the midgut. The midgut loop and dorsal mesentery elongate rapidly these structures temporarily herniate into the extraembryonic coelom within the umbilical cord. The cranial limb elongates rapidly whereas the caudal limb grows the caecal bud. The herniated contents rotate 90 degrees anticlockwise and once it return into the peritoneal cavity the proximal part of the limb passes posterior to the SMA. The small bowel occupies the right side of the coelomic cavity and the colon returns rotating a further 180 degrees to the right [4,5]. The definitive peritoneal attachments occur during fetal life. As a result of this, there are three types of intestinal malrotation as classified by Stringer et al.: Type 1 - Nonrotation, Type 2 - Duodenal malrotation 180 degrees, and Type 3 - Duodenal and caecal malrotation with SMA anterior to the transverse colon [6]. In addition, midgut volvulus along the SMA axis occurs due to the narrow mesenteric base and the presence of Ladd’s bands; in normal embryology this is prevented by the fetal formation of a broad mesenteric root from the duodenojejunal flexure to the ileocaecal junction.

Intestinal malrotation is largely a condition that manifests in infants with 90% occurring within the first year of life and 80% within the first month [7]. They classically present with bilious vomiting and abdominal pain in a previously well infant, however, nonbilious vomiting does not rule out the differential of intestinal obstruction secondary to midgut volvulus. Despite this known infantile predilection, increasingly is the diagnosis in older children and as in our case, adults with an incidence of 0.2% to 0.5% [8]. One recent meta-analysis has noted the most common age range of presentation being 25-40 years (41.3%), with the average being 38.9 years [3]. Presentation of this pathology may be asymptomatic where it is found incidentally during imaging or laparotomy for another condition, acute presentation with volvulus, or chronic symptoms such as intermittent abdominal pain, nausea vomiting over years. It is important to note that 90% of patients had a history keeping with chronic symptomatology. Contributing to our patient’s volvulus may have been as a result of his weight loss which may have reduced overall fat of the mesentery allowing for easier rotation and subsequent volvulus, an associated reported in 2.6% of cases [3,9].

Investigation of symptoms either acute or chronic in adults, unlike in children usually involves CECT of the abdomen to identify an offending etiology. Findings in keep with intestinal malrotation include the abnormal SMA/SMV relationship (normally SMV lies to the right of the SMA), absence of a retro mesenteric duodenum, mesenteric whirl sign, all of which our patient had. Other investigations such as plain X-ray film, upper gastrointestinal fluoroscopic study, and abdominal ultrasound are nonspecific but can aid in diagnosis of small bowel obstruction [6,10]. Regardless of age, the corrective operative procedure entails Ladd’s procedure which involves the re-rotation of the volvulus, division of Ladd’s bands, broadening of the small bowel mesentery, incidental appendicectomy, and placement of bowel in a non-rotated position [4,6]. Further to this procedure, which was done for our patient, a coexisting internal hernia was also identified. The association of internal hernias with congenital intestinal malrotation with simultaneous internal hernia has been cited in the literature with para-duodenal hernias being the most common (53%) (left > right) [11-14], however, in our case we present a patient with a trans-meso-colic hernia (prevalence ranging from 2% to 10%) [15,16] of the ascending colon. Trans-meso-colic hernias are most often represented by those traversing the transverse mesocolon with those of the ascending colon being exceedingly rare, with only three noted in the literature [17].

Though the standard of care for acute cases of volvulus secondary to intestinal malrotation is established in Ladd’s procedure, there is controversy surrounding the management of incidental malrotation and patients with chronic symptoms. It is thought that the cause of chronic or atypical symptoms may arise secondary to primary intestinal motility pathology but another school of thought considers the use of diagnostic laparoscopy to assess the patient’s anatomy and risk for volvulus.

## Conclusions

Although the presentation of adult intestinal malrotation is rare, clinicians should be wary of it and assess patients with chronic symptoms for early evaluation to prevent morbidity associated with acute presentations. It is also important to recall the association of internal hernias with malrotation and adequately assess for and manage the presence of internal hernias. Future studies should aim to quell the ongoing controversies in management.
